# Electrospinning and emerging healthcare and medicine possibilities

**DOI:** 10.1063/5.0012309

**Published:** 2020-07-14

**Authors:** Ziqian Liu, Seeram Ramakrishna, Xiaoling Liu

**Affiliations:** 1 Department of Mechanical, Materials and Manufacturing, The University of Nottingham Ningbo China, Ningbo 315100, China; 2 Center for Nanofibers and Nanotechnology, Department of Mechanical Engineering, National University of Singapore, Singapore 117581, Singapore

## Abstract

Electrospinning forms fibers from either an electrically charged polymer solution or polymer melt. Over the past decades, it has become a simple and versatile method for nanofiber production. Hence, it has been explored in many different applications. Commonly used electrospinning assembles fibers from polymer solutions in various solvents, known as solution electrospinning, while melt and near-field electrospinning techniques enhance the versatility of electrospinning. Adaption of additive manufacturing strategy to electrospinning permits precise fiber deposition and predefining pattern construction. This manuscript critically presents the potential of electrospun nanofibers in healthcare applications. Research community drew impetus from the similarity of electrospun nanofibers to the morphology and mechanical properties of fibrous extracellular matrices (ECM) of natural human tissues. Electrospun nanofibrous scaffolds act as ECM analogs for specific tissue cells, stem cells, and tumor cells to realize tissue regeneration, stem cell differentiation, and *in vitro* tumor model construction. The large surface-to-volume ratio of electrospun nanofibers offers a considerable number of bioactive agents binding sites, which makes it a promising candidate for a number of biomedical applications. The applications of electrospinning in regenerative medicine, tissue engineering, controlled drug delivery, biosensors, and cancer diagnosis are elaborated. Electrospun nanofiber incorporations in medical device coating, *in vitro* 3D cancer model, and filtration membrane are also discussed.

## INTRODUCTION

**FIG. 1.**
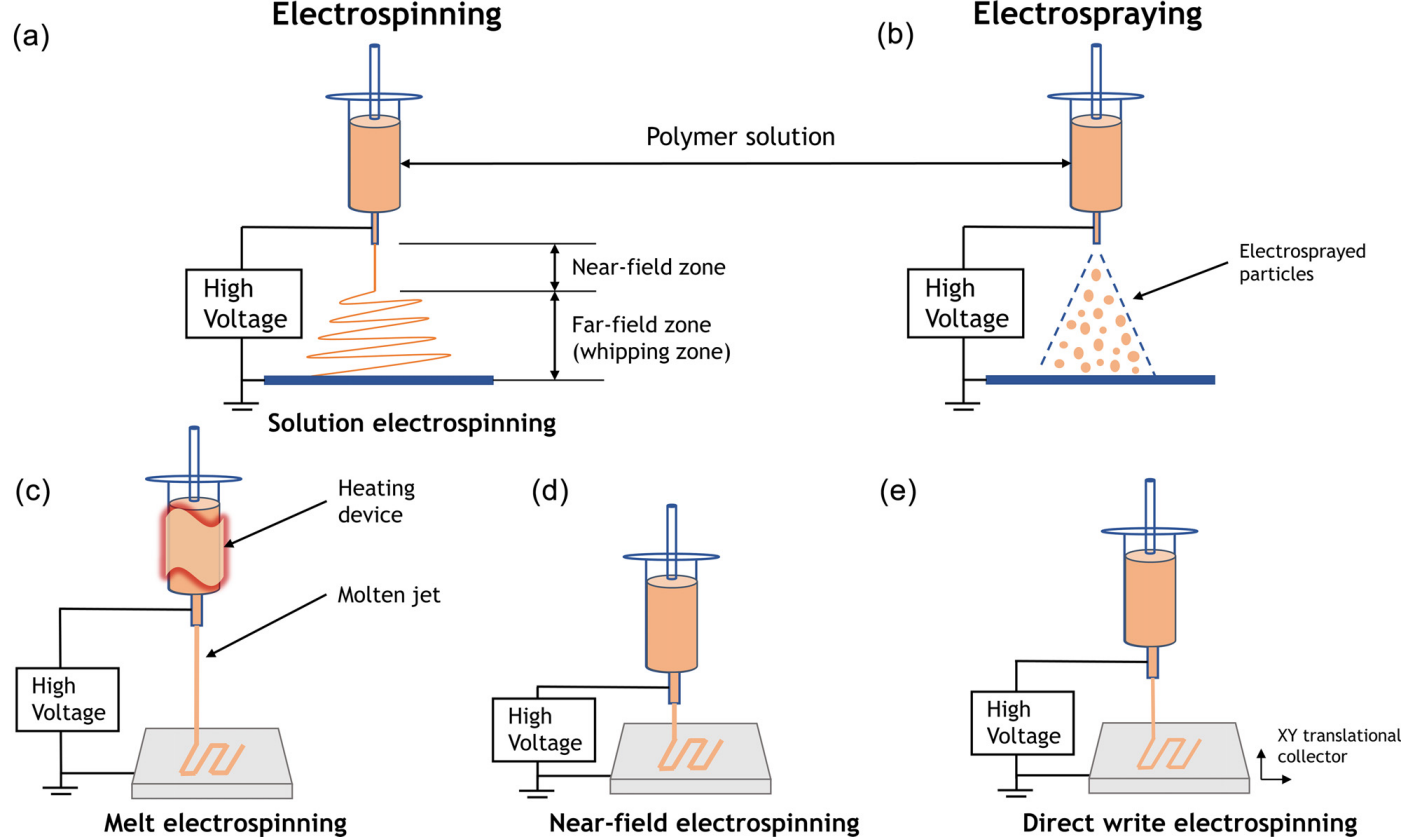
Schematic illustrations of (a) electrospinning and (b) electrospraying. The charged jet can be kept in a continuous form to produce fibers in electrospinning, whereas it breaks into droplets to form particles in electrospraying. During electrospinning, the ejected jet initially follows a straight line in the near-field zone and undergoes stretching and thinning upon whipping motions in the far-field zone. (c) Schematic illustration of melt electrospinning. Unlike conventional solution electrospinning, a heating device is attached to maintain a molten jet in melt electrospinning. Normally, the jet travels in a straight line and generates micrometer scale fibers. (d) Schematic illustration of near-field electrospinning. The jet deposited on the collector within the straight segment, which shows higher spatial control of fiber placement but larger fiber diameter. (e) Schematic illustration of direct write electrospinning that integrates AM concept to electrospinning. A translational collector is used for predefined pattern construction.

As reported by “Research and Markets,” the global market for nanofibers can reach 1 billion U.S. dollars by the end of 2021.^1^ Electrospinning is a simple and versatile process to fabricate micrometer and nanometer scale thickness fibers that contribute to the emerging nanotechnology field. In simple terms, the electrospinning process relies on an electrohydrodynamic principle that a highly electrified polymer solution or melt is forced to stretch and elongate into fibers [Fig. 1(a)]. Electrospraying involves applying high voltage to liquid jets as well, whereas particles are collected instead of fibers [Fig. 1(b)].

In solution electrospinning where a polymer solution is elongated and thinned by whipping effect, quick evaporation of solvents results in solidification of the polymer jet and nanofiber deposition, whereas in melt electrospinning [Fig. 1(c)], polymers remain in a molten state that exhibit lower electrical conductivity, higher viscosity, and lower density of surface charge than polymer solutions. The solidification of molten jets relies on the heat transfer between the jet and surrounding medium. Such rapid solidification further suppresses the whipping effect and requires stronger electrostatic repulsion to overcome the viscoelastic force.^2^ Therefore, the electrostatic force mainly contributes to thinning of the molten jet, which is not as sufficient as solution electrospinning with whipping effect and solvent evaporation. The diameter of melt electrospun fibers is typically on the micrometer scale, while the jet travels in a straight path before deposition and solidification, allowing better fiber placement control. Since a heating device is included in the melt electrospinning setup, thermoset polymers, thermally unstable polymers, and some bioactive molecules cannot be incorporated. Conventional electrospinning is usually conducted in the far-field model where the distance between the spinneret and collector ranges from 5 to 15 cm with a high applied voltage (10–20 kV).^2^ By reducing that distance to 500 *μ*m–1 cm,^2,3^ near-field electrospinning can be obtained [Fig. 1(d)]. The electric field will be highly concentrated within such short distance that permits substantially reduced applied voltage to several hundred volts (normally 0.6–3 kV).^3^ The whipping instability is dampened in near-field electrospinning, and the jet is deposited on the collector within straight segments. Similarly, like melt electrospinning, near-field electrospinning allows precisely spatial control of fiber deposition together with fiber diameters on the micrometer scale. Yet with lower applied voltage, near-field electrospinning is more suitable than melt electrospinning for bioactive molecules accommodation.

Recently, researchers have adopted additive manufacturing (AM) concept to electrospinning for more accurate control of fiber deposition.^4–6^ AM enables fabrication of 3D constructs with customized geometry and structure, whereas the spatial resolution is quite limited. Direct write electrospinning [Fig. 1(e)] is an integration of electrospinning and AM that combines the nanofibrous characteristics of electrospinning and accurate designing potential of AM. In direct write electrospinning, the jet is focused to travel in a straight line via auxiliary electrodes, melt electrospinning, or near-field electrospinning. Together with predefining translational movement of the collector, 3D constructs with designed patterns and accurately controlled features such as pore size^7^ can be produced.

Initial applications of electrospinning emerged in air filtration and personal protection purposes. Subsequently, efforts are made for diverse applications in medicine and healthcare, water treatment, damage resistant composites, light weight buildings and construction, mitigation of noise pollution, energy generation and storage, photonics, electronics, and wearables. This paper focuses on medicine and healthcare applications.

Bioengineering and biomedical engineering research community drew impetus from the similarity of electrospun nanofibers to the morphology and mechanical properties of fibrous extracellular matrices, extracellular matrix (ECM) of natural human tissues [Table I (Refs. 2, 8, and 9)]. ECM is a collection of extracellular molecules secreted by cells that provide mechanical support and biochemical cues to surrounding cells and tissue.^2^ It is a complex and heterogeneous network with tissue-specific characteristics. The highly porous structure of ECM allows nutrients and oxygen diffusion and transport. Adequate pore size is critical to support cell–cell and cell-matrix interactions. Collagen is the most abundant fibrous protein in native ECM, which offers structural support and topographic guidance through specific orientations to surrounding cells and facilitate cell–cell interactions. Collagen exists as nanofibers with a diameter of 50–500 nm and accounts for the nanofibrous structure of ECM with specific fiber alignment. Biomolecules residing in the ECM provide intrinsic biochemical cues for regulating cellular functions as well as cell-matrix interaction. Mechanical properties such as stiffness of the ECM can affect cellular activities including adhesion, migration, proliferation, and differentiation. For example, neural stem cells cultured on a stiffer matrix (1–10 kPa) showed glial differentiation, while exhibited neural differentiation on a softer matrix (100–500 Pa).^10^ Similarly, electrospun nanofibers can assemble into a nanofibrous network with tailorable porosity and pore size. The diameter of nanofibers can be altered accordingly as well. Through various electrospinning setups such as different collectors design, the fiber orientation can be customized. Electrospinning of various materials blends leads to optimal mechanical properties of electrospun fibers that maintain the integrity and match with native ECM.

**TABLE I.** Similarities between electrospun fibers and fibrous ECM.

Characteristics	Natural ECM	Electrospun fibers
Diameter of fibrous components	50–500 nm (collagen fibers)^2^	Tens to hundreds of nanometers
Porosity and pore size	Highly interconnected pores	Highly porous
	Tissue-specific	
	e.g., Ref. 8 Neovascularization: 5 *μ*m	Interconnected pores
	Fibroblast ingrowth: 5–15 *μ*m	Tailorable
	Bone regeneration: 200–350 *μ*m	
	Skin regeneration: 20–125 *μ*m	
Mechanical properties	Tissue-specific	Tailorable
	e.g., Ref. 9 Cancellous bone: 0.4 GPa (modulus); 7.4 MPa (tensile strength)	Vary across materials selection, porosity control and fiber orientation
	Articular cartilage:10.5 MPa (modulus); 27.5 MPa (tensile strength)	
	Skin: 0.1–0.2 MPa (modulus); 7.6 MPa (tensile strength)	
Physical architecture	Tissue-specific	Tailorable
	E.g. Skin: basketweave-like pattern of collagen fibers	
	Tendon: parallelly aligned collagen fibers	

**FIG. 2.**
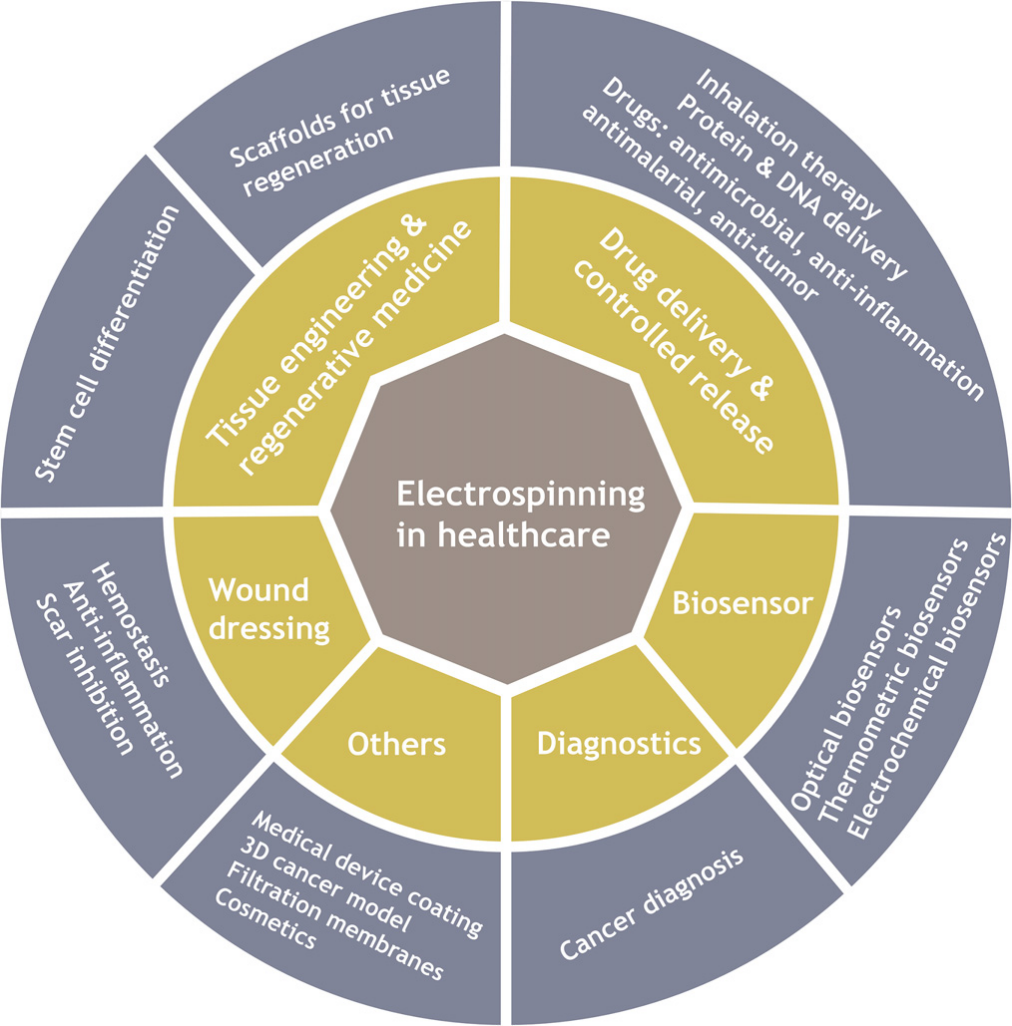
Applications of electrospinning in healthcare.

**FIG. 3.**
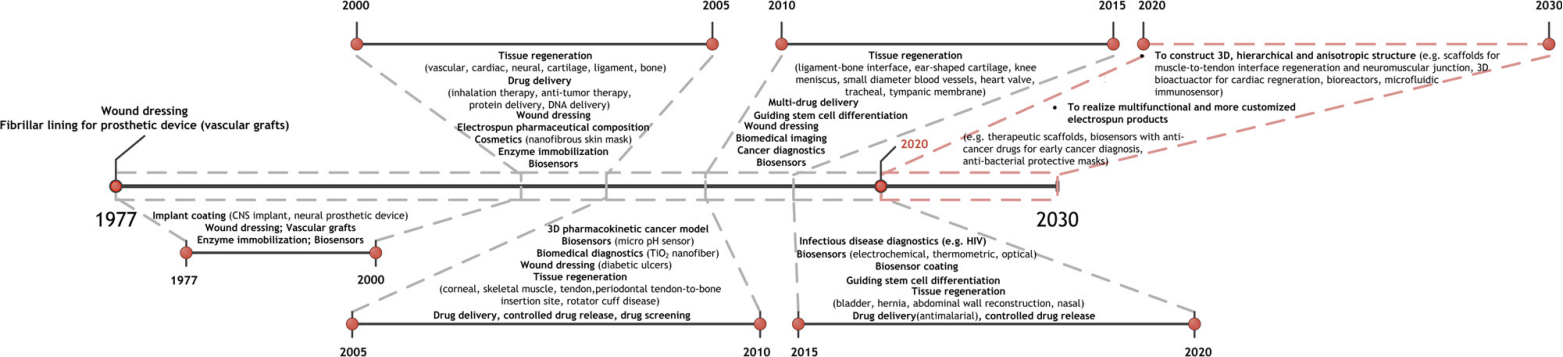
Development of electrospinning in biomedical applications. Electrospun nanofibers have been utilized in biomedical applications mainly as wound dressing and implant coating since 1977. There were broad applications of electrospinning in healthcare in the past two decades. From 2000 to 2020, the key applications of electrospinning in healthcare are summarized and presented at 5-year intervals. From 2020 to 2030, two future trends of applying electrospinning in healthcare are suggested with examples. CNS implant: central nervous system implant.

Nanofibers with relatively large surface-to-volume ratio and nanofibrous structure with high porosity make electrospun products potential for tissue engineering and regenerative medicine, drug delivery, biosensors, diagnostics, etc. (Fig. 2). The first patented electrospun product in biomedical applications is a wound dressing mat by Martin *et al.* in 1977.^11^ Since then, electrospinning has been widely explored for healthcare applications specifically over the past two decades (Fig. 3). Herein this review, we described recent advances related to biomedical applications of electrospinning through representative examples. We mainly focus on electrospun nanofibers in tissue engineering and regenerative medicine, drug delivery, biosensors, and cancer diagnosis. Other applications including medical device coating, *in vitro* 3D cancer models, viral and microbial resistant surgical masks, respirators, personal protective equipment, and filtration membranes related to the coronavirus disease 2019 (COVID-19) outbreak are discussed as well. Current challenges in each application are mentioned along with future perspectives.

## TISSUE ENGINEERING AND REGENERATIVE MEDICINE

**FIG. 4.**
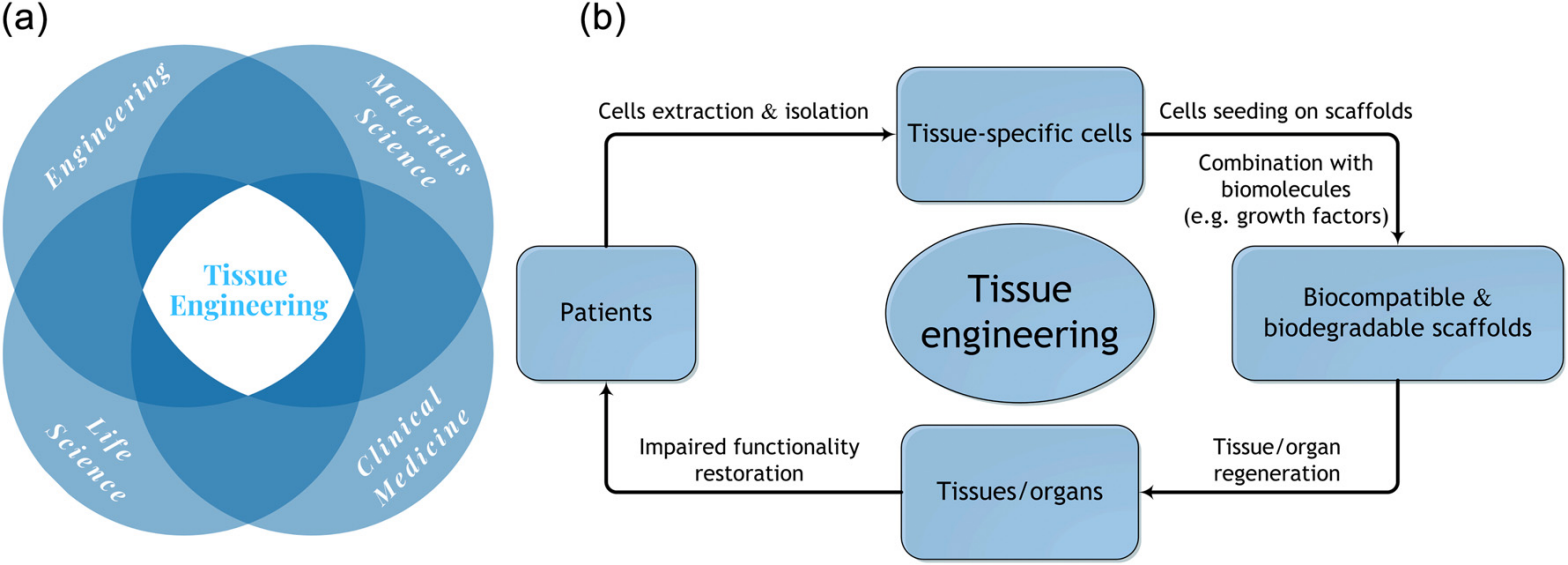
Basic concept of tissue engineering approach for tissue regeneration. (a) Tissue engineering is a highly multidisciplinary field that recruits experts from engineering, materials science, life science, and clinical medicine. (b) In tissue engineering, biocompatible scaffolds act as a temporary template for tissue-specific cell growth and proliferation, and are occasionally incorporated with biomolecules for enhanced cell regulation and tissue regeneration. Upon implantation of the engineered tissue, scaffolds will gradually degrade leaving regenerated tissues or organs with restored functionality.

Tissue engineering, as first fully illustrated by Langer and Vacanti,^12^ is a highly multidisciplinary field for damaged tissue regeneration that accommodates cells into porous scaffolds made of biomaterials and guide their growth to new tissue^13,14^ (Fig. 4). Electrospinning has been widely explored for nanofibrous scaffolds fabrication and achieve promising replication of native ECM in terms of composition and architecture, whereas the composition and structure of ECM are tissue specific leading to specific requirements in different applications.^15^ Applications of conventional electrospun scaffolds in tissue engineering are limited because of the mechanical incompliance between scaffolds and native ECM such as in bone and cardiac regeneration, inefficient replication of complex and anisotropic structure such as in tendon and cartilage and tissue-to-tissue interface, and difficulty in providing specific functionality such as electrochemical stimulation to neural and cardiac tissue, anti-thrombogenicity of vascular grafts, and scar inhibition in skin regeneration.

Recent electrospun scaffolds in tissue engineering and regenerative medicine could combine various guidance to address aforementioned problems via a synergistic effect on stem cell differentiation [Table II (Refs. 6 and 16–34)] and tissue regeneration. Basically, topographical and biomechanical cues can be generated via scaffold design and configurations, biological cues can be accomplished by bioactive agents and/or biomolecules incorporation, and electrochemical cues are attained through material selection, fiber chemistry, thickness, and porosity control as needed. Fibrous proteins assemble a 3D network in ECM with specific fiber orientation varying across tissues. Compared to conventional 2D electrospun scaffolds, 3D scaffolds are favored in resembling natural ECM. Electrospinning in specific tissue engineering applications as the potential solution to current limitations is discussed in the section titled Tissue Engineering and Regenerative Medicine.

**TABLE II.** Examples of electrospinning for stem cell differentiation. MSCs: mesenchymal stem cells; iPSCs: induced pluripotent stem cells; BMP-2 peptide: bone morphogenic protein-2.

Electrospun scaffolds features	Stem cell types	Stem cell differentiation
Topographical and mechanical cues		
Uniaxially aligned nanofibers^16,17,27^	Adipose derived MSCs iPSCs derived MSCs	Tenogenic differentiation
	Human fetal osteoblasts	Osteogenic differentiation
	Bone marrow derived MSCs	Neural differentiation
Uniaxially aligned yarns^28^	Adipose derived MSCs	Anisotropic soft tissue differentiation
Orthogonal layers^29^	Bone marrow derived MSCs	Osteoblastic differentiation
Coiled nanofibers^30^	Bone marrow derived MSCs	Mild myofibroblastic differentiation
Honeycomb-compartmented monolayer^31^	iPSCs	Cardiac differentiation
Netslike nanofibrous mesh^32^	Bone mesenchymal stromal cells	Osteogenic differentiation
Zonal organized nanofibers^6^	Mesenchymal stromal cells	Chondrogenic differentiation
Higher degree of roughness	Mesenchymal stromal cells	Osteogenic differentiation
Lower degree of roughness^33,34^		Chondrogenic differentiation
Lower stiffness^18^	Smooth muscle cells	Contractile phenotype
Dynamic mechanical stimulation^19^	Adipose derived MSCs	Tenogenic differentiation
Electrochemical cues		
Electrical pulse application^20^	Cardiovascular disease specific iPSCs	Cardiomyocytes
Piezoelectric scaffold^21^	Bone marrow derived MSCs	Osteogenic differentiation (high voltage)
		Chondrogenic differentiation (low voltage)
Biological cues		
Hemin doping^22^	iPSCs derived neural stem cells	Neural differentiation
Retinoic acid induction^23^	Chorion derived MSCs	Neural differentiation
Peptide decoration^24^	Human PSCs	Osteogenic differentiation
BMP-2 peptide^25^	Adipose derived MSCs	Osteogenic differentiation
Co-culture with chondrocytes^26^	Bone marrow derived MSCs	Chondrogenic differentiation

### Electrospinning in bone regeneration

Mechanical properties incompliance between electrospun scaffolds and native bones is a common problem. Polymers are the most commonly electrospun materials, yet exhibit lower modulus than native hard tissue in general.^9^ The ideal scaffold should offer sufficient mechanical support during bone regeneration and biochemical guidance to induce osteogenesis for healing acceleration. A sponge-like 3D nanofibrous silk fibroin/poly(ε-caprolactone) (PCL) scaffold was mineralized and immobilized with BMP-2 peptide.^25^ The mineralization improved the compressive modulus of scaffolds significantly (468.5 ± 48.7 kPa vs 109.3 ± 21.8 kPa). However, such modulus is much lower than that of native cancellous bones of 0.4 GPa.^9^ The authors suggested that those sponge-like scaffolds were potential to calvarial defects. Since the major contents in the bone are type I collagen fibrils and hydroxyapatite nanoparticles, for bone regeneration, biocompatible and biodegradable polymeric scaffolds are always reinforced with inorganic substance such as hydroxyapatite, bioactive glass, and silica. An electrospun scaffold out of silk fibroin/PVA/58S bioglass was developed for bone regeneration.^35^ The addition of bioactive glass significantly improved the mechanical properties of scaffolds with a raised Young's modulus from 293 ± 64 MPa to 655 ± 151 MPa. Considering relatively high modulus of ceramics and metals, incorporation of such stiffer materials into a polymeric matrix or developing polymer-free ceramic scaffolds for hard tissue regeneration is expected.

### Electrospinning in cartilage regeneration

Unlike bone, the major components in cartilage are type II collagen and proteoglycan. There is a zonal organization and distribution of collagen in cartilaginous ECM that the content decreases from the superficial zone to the deep zone. Collagen fibrils are aligned parallel to the articular surface in the superficial zone, while they are oriented perpendicular to the articular surface in the middle and deep zones. For 2D planar mats, the ongoing challenge is to achieve complete cell infiltration through the defects. A 3D nanofibrous scaffold with hierarchical architecture, sufficient compressive strength, and highly interconnected pores is more ideal. Chen *et al.* combined direct writing with solution electrospinning for hierarchical scaffolds fabrication mimicking the zonal organization of articular cartilage.^6^ The 3D multiscale fibrous scaffolds promoted chondrogenic differentiation of hMSCs and directed tissue organization in a zone-dependent way.

### Electrospinning in tendon and ligament regeneration

Collagen fibers are closely packed in parallel arrays in tendon and ligament. Thus, uniaxially aligned nanofibrous mats are widely applied to tendon and ligament regeneration. Uniaxial aligned nanofibers can be easily collected using a rotating collector or extra parallel electrodes. Adipose derived MSCs and iPSC derived MSCs show tenogenic differentiation on uniaxial nanofibers. However, the bulk of tendon and ligament tissue present highly anisotropic structures. 3D scaffolds with braided, woven, or knitted yarn networks are more favored compared to 2D mats. When including hydrogels into the nanofibrous matrix, it can benefit biomolecule and cell encapsulation. For example, unidirectional PCL nanofibers were once coated with chitosan/hyaluronic acid hydrogel for ligament regeneration.^36^

### Electrospinning in tissue-to-tissue interface regeneration

Currently, regeneration of tissue-to-tissue interface remains challenging. Those interfaces contain soft-to-hard interfaces and soft-to-soft interfaces. In the junctions between a soft matrix and a hard matrix (e.g., tendon/ligament-to-bone, cartilage-to-bone), there are gradual variations in matrix composition, architecture, mineral content, and significant difference in mechanical properties.^37^ An ideal scaffold should contain a hierarchical structure that allows a transmission in structure and mechanical stress between two mechanically differed tissues. Spatially organized nanofiber structure, material composition with graded distribution, and bioactive agents can attribute to a specialized scaffold for regeneration of such interfaces. A nanofibrous mat with “aligned-to-random” nanofibers was developed to mimic the graded structure in the tendon-to-bone interface.^38^ The aligned nanofibers represented highly aligned collagen fibers and tendon, while the random portion mimicked the less ordered collagen fibers in bones. A dual-layer organic/inorganic nanofibrous scaffold was fabricated to recapitulate the gradient mineral content within the tendon-to-bone interface.^39^ The PLLA/nanohydroxyapatite (nHA) nanofibrous mat was synthesized via electrospinning of nHA on the top of the PLLA electrospun mat, which resembled the mineralized and non-mineralized fibrocartilage in the interface, respectively. For cartilage-to-bone interface regeneration, nanofibrous scaffolds are always combined with hydrogels since cartilage is a resilient tissue. Mohan *et al.* designed a 3D hybrid scaffold that assembled PCL nanofibers with gradients of chondroitin sulfate and bioactive glass into hydrogel.^40^ The chondroitin sulfate addition promoted glycosaminoglycan-enriched ECM secretion by chondrocytes mimicking the hyaline cartilage, whereas the bioactive glass content enhanced mineralized ECM formation.

Muscle-to-tendon interface and interface at the neuromuscular junction are two typical soft-to-soft tissue interfaces. The myotendinous junction connects dense collagen fibers in tendon to softer muscle fibers. The main difficulty in regeneration of myotendinous junction is a comparable stiffness transition to native junctions. For example, Ladd *et al.* developed PCL/poly (lactic acid) (PLA) nanofibrous scaffolds which followed the mechanical trend of native myotendinous junctions.^41^ However, the ratio of tendon to muscle stiffness was 6, which is far from the natural ratio in the range of 179–37 000.^42^ Studies on neuromuscular junction remodeling are quite limited. The natural neuromuscular junction is essential to support native functionality of motor neurons and skeletal muscles. The scaffolds should recapitulate the innervation in living skeletal muscles and restore the response of muscles to neurotransmitters.^42^ Culturing stem cells on the scaffolds and guiding them into desired differentiation such as tenogenic, myofibroblastic, and neural differentiation may help with the interface reconstruction.

### Electrospinning in cardiac regeneration

It is critical to maintain sufficient mechanical strength in the cardiac tissue to sustain myocardium contraction and relaxation. The perimysial fibers in myocardium exhibit a coiled structure and contribute to the interwoven structure of myocardium. Complete replication of such anisotropic organization and mechanical capability of cardiac tissue remains challenging. Liu *et al.* developed honeycomb-patterned scaffolds for better mimicking the myocardium architecture.^43^ The beating rate of cardiomyocytes on patterned scaffolds showed a comparable value to adult or neonatal rats. A study investigated coiled PCL nanofibers together with gold nanoparticles to restore the myocardium functionality.^44^ The scaffold design is mainly focused on planar scaffolds, which have limited effect on myocardium maturation.^45^ A 3D scaffold that replicates the architecture, the mechanical properties, and the native function of cardiac tissue is highly desired in the future. Since the heart wall is a multi-layered structure, scaffolds composing of multilayers or a yarn network are suggested for more efficient tissue regeneration.^2^ Each layer can be individually controlled over the nanofiber orientation and layer pattern. Thus, the interwoven and anisotropic structure of native cardiac tissue can be better emulated. To further restore the functionality of the myocardium, 3D electrospun scaffolds integrated with electrochemical cues are favored. Wang *et al.* developed a 3D bioactuator in a tubular shape by loading cardiomyocytes on conductive PLA/PANi nanofibers, which combined topographic, biological, and electrochemical cues together.^46^ Cardiomyocytes on such 3D scaffolds showed higher beating frequency than those cultured on planar scaffolds.

### Electrospinning in neural regeneration

Both uniaxially and radially aligned nanofibers have been demonstrated to be effective in neural regeneration. Bone marrow derived MSCs can differentiate into Schwann cells on uniaxially oriented PCL nanofibers.^27^ Radially aligned nanofibers are attractive for spinal cord injury repair, which recruits cells from the central canal region to lesion site. Li *et al.* developed radially aligned electrospun collagen/PCL mats with a circular gradient of biomolecules incorporation.^47^ Such scaffolds directed and promoted neural stem cells migrated from the periphery to center along nanofibers. However, planar scaffolds are mainly used in *in vitro* neural tissue regeneration studies. A nerve guidance conduit (NGC) with a complex, multitubular structure is widely used in *in vivo* studies. Currently, including all the topographic and biochemical characteristics of the native nerve tissue in the NGC design remains challenging. The size of intraluminal microchannels in NGC can be controlled via sacrificial templates such as sucrose fibers.^48^ With a controllable channel, intraluminal fillers can be introduced to obtain additional topographical cues. Poly(lactic-co-glycolic) acid (PLGA) unidirectionally aligned nanofibers with laminin coating have been used as fillers within NGC, which provides topographic and biological guidance to nerve tissue regeneration simultaneously.^49^ Piezoelectric polymers and conductive polymers can provide electrical stimulation to cells. A conductive polypyrrole (PPy)/silk fibroin NGC was fabricated by 3D printing and electrospinning.^50^ Unidirectional PPy/silk fibroin fibers were 3D printed and incorporated as intraluminal fillers. A dual-layer of electrospun aligned and random silk fibroin nanofibers assembled the shell of NGC. The topographic and electrical cues of such NGCs allowed proliferation of Schwann cells and *in vivo* axonal regeneration. Further study can focus on integrating biological, topographic, and electrochemical cues in nanofibers for better neural regeneration due to the synergistic effect of those guidance.

### Electrospinning in vascular regeneration

There are commercially available vascular grafts for large-size blood vessels regeneration, yet studies of regenerating blood vessels of small diameter (i.e., <6 mm) are quite limited. Unlike the grafts for large-diameter arteries, the major concern for small-diameter vascular grafts is maintaining the lumen patency.^51^ Graft occlusion typically occurs in small-size blood vessels due to acute thrombosis and intimal hyperplasia, which significantly impairs vascular grafts function. Construction of a non-thrombogenic scaffold surface and achieving rapid endothelization within the electrospun scaffolds are both critical in avoiding thrombosis. There is limited research investigating the scaffold surface configuration for anti-thrombosis. Current approaches are incorporation of biomolecules into nanofibers such as heparin for anticoagulation and accelerating endothelization within the grafts. Surface modified with biomolecules (e.g., vascular endothelial growth factors) through covalent bonding or the core-sheath structure to promote endothelial cells recruitment^52^ and local delivery of miRNA to modulate the endothelial cell phenotype have been both investigated.^53^ Other approaches rely on direct pre-seeding of endothelial cells or endothelial progenitor cells on electrospun grafts.^54^ Maintaining a contractile phenotype of smooth muscle cells (SMCs) is critical in minimizing intimal hyperplasia. Highly aligned PCL/hyaluronan nanofibers promoted higher contractile gene expression in SMCs.^55^ It is worth to note that intimal hyperplasia occurs in all vascular grafts with different degrees due to blood flow profiles, which is independent of scaffold materials,^51^ while compliance mismatch between the scaffolds and native vessels partially results in intimal hyperplasia. Developing a vascular graft to obtain mechanical properties consistent with native vessels is of great importance.

### Electrospinning in skin regeneration

In native ECM, the fibrous structure always exhibits a more complex architecture other than simple unidirectional alignment. Collagen fibrils in skin tissue show a mesh-like or a basketweave-like pattern. Therefore, scaffolds with crossed nanofibers showed higher keratinocytes and fibroblasts migration rate, thus better wound healing performance than either random or unidirectionally aligned nanofibers.^56^ A highly porous cotton-wool-like PCL/chitosan scaffold was developed using emulsion electrospinning and achieved accelerated full-thickness wound healing in 3 weeks *in vivo.*^57^ One major concern for wound healing is scar inhibition, which is a long-lasting obstacle in clinical studies. Abnormal fibroblast proliferation and subsequent collagen deposition lead to scar formation. Several cell signaling molecules including basic fibroblast growth factor (bFGF), transforming growth factor-β1 (TGF-β1), and ginsenoside-Rg3 have been studied to address it. Ginsenoside-Rg3 and bFGF can promote the normal function of fibroblast; thus, they have been introduced to PLGA nanofibers by blending electrospinning and surface immobilization,^58^ whereas TGF-β1 signaling would promote fibroblast abnormal proliferation. TGF-β1 inhibitors were loaded in PCL/gelatin nanofibers via blend electrospinning for hypertrophic scarring inhibition.^59^ To prevent scar formation, the effect of nanofibers on the cellular signaling mechanisms and biochemical pathways should be elucidated. Whether the nanofiber features such as fiber diameter and fiber alignment would influence the scar inhibition remains to be determined.

## DRUG DELIVERY AND CONTROLLED RELEASE

**FIG. 5.**
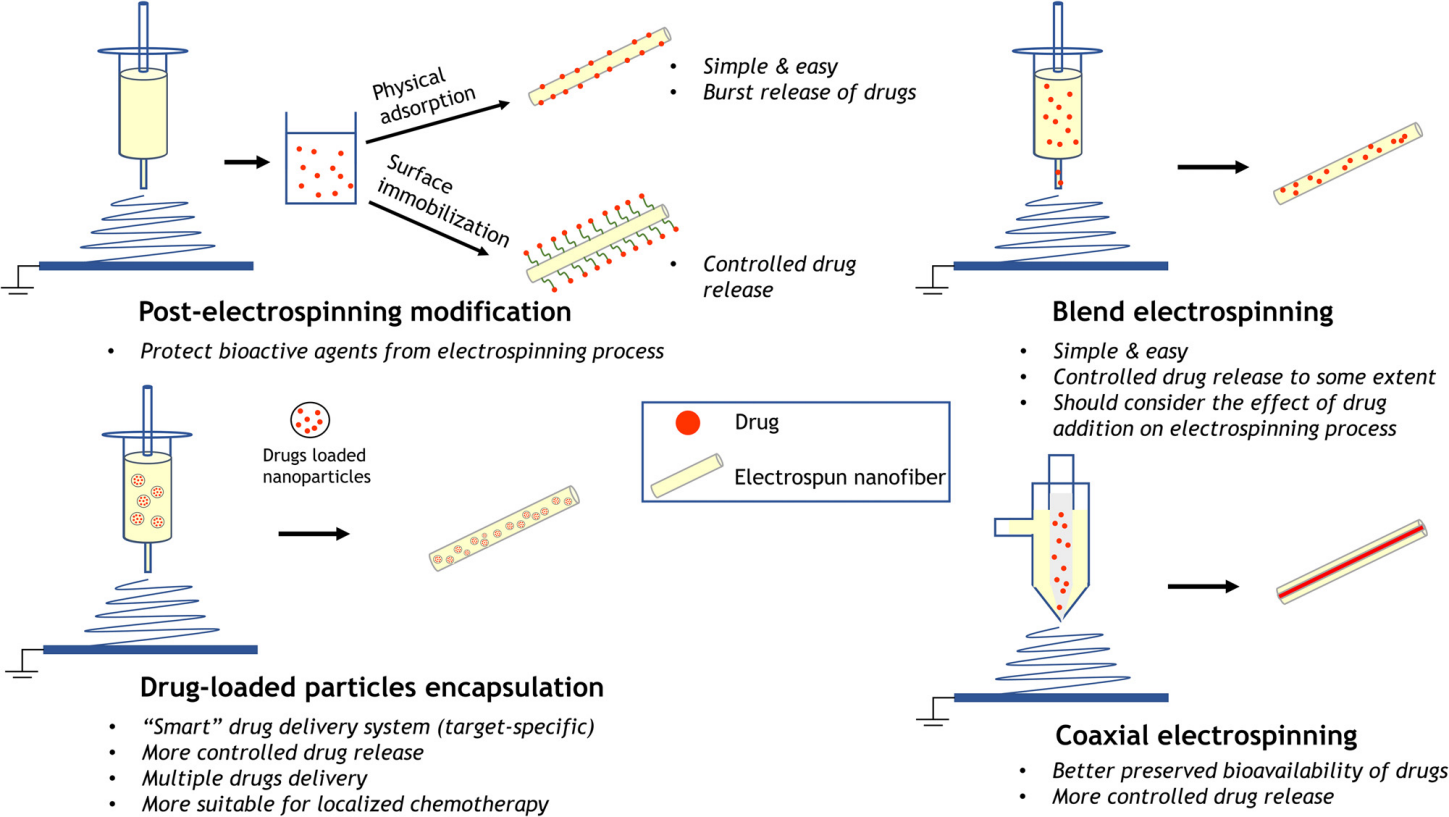
Common approaches of loading drugs into electrospun nanofibers. Post-electrospinning modifications including simple physical adsorption and surface immobilization for more controlled drug release. Blend electrospinning and coaxial electrospinning allow drug encapsulation to as-spun nanofibers. Loading drugs into particles followed by particle incorporation to nanofibers permits a more versatile drug delivery system by tailoring the characteristics of both nanofibers and particles. Characteristics of each approach are presented.

The principle of using polymer nanofibers as drug carriers is that the dissolution rate of drugs increases with the increase in surface area of both drugs and carriers.^60^ The inherent higher surface-to-volume ratio of electrospun nanofibers affords high drug loading capacity and efficiency. Electrospun nanofibers can achieve controlled drug release with better preserved bioavailability by different drug encapsulation designs. Typical drug loading strategies include post-electrospinning modification, blend electrospinning, coaxial electrospinning, and nanoparticles encapsulation (Fig. 5). Different drug loading strategies lead to different interactions between drugs and nanofibers, which results in different drug releasing kinetics. Initial burst release typically occurs in drug loading avenues. Practically, initial release of specific drugs in clinic can benefit the anti-inflammatory effect in the early stage. It would be more favorable to secure controlled and sustained drug release in complex scenarios. The localized and target-specific delivery of anti-cancer drugs can further eliminate potential damage of chemotherapy to surrounding tissue. Potential strategies to achieve controlled drug release with preserved bioactivity are discussed in the section titled Drug Delivery and Controlled Release.

Physical adsorption is the simplest way to introduce drugs to the electrospun matrix, which is prone to an initial burst release and inefficient in sustained drug release.^61^ Since the post-electrospinning concept can prevent bioactive agents from destabilization and denaturation during the electrospinning process, research on more controlled release with surface localized drugs is highly encouraged. One strategy is surface immobilization of drugs to nanofibers and by controlling the degradation rate of a specific conjugation linker to achieve controlled drugs release. For instance, it is reported that there is an inverse correlation between matrix-metalloproteinase (MMP) levels and diabetic ulcer wound healing rate.^62^ A complex of linear PEI and DNA was conjugated to a PCL/polyethylene glycol (PEG) scaffold via an MMP cleavable peptide linker for diabetic ulcer healing.^63^ Compared to direct immobilization of DNA to scaffolds, the modified conjugating system showed an MMP dependent release profile.

Blend electrospinning and emulsion electrospinning of bioactive drugs and polymers are two typical strategies to incorporate drugs in as-spun nanofibers. Electrospinning of drug and polymer blends with a hand-hold device allows for direct deposition of drug loaded fibrous matrix to the wound site. However, one thing worth noted is that the addition of drugs could affect the properties of electrospinning solution such as solution conductivity and viscosity. When loading meloxicam in PVA solutions, the solution viscosity was elevated and, in turn, resulted in thicker fibers as reported.^64^ Besides, improving the drug-polymer compatibility is considered worth for more controlled drug release. Otherwise, drugs tend to accumulate on the surface of nanofibers leading to a burst release.^65^ Zeng *et al.* investigated different release kinetics between doxorubicin base and doxorubicin HCl in electrospun PLLA nanofibers.^66^ Results revealed that there was a 70% burst release of doxorubicin HCl due to a rapid wash-off from the nanofiber surface where it got accumulated. In contrast, doxorubicin base only showed a 20% burst release since it embedded into the fibers other than residing on the surface.

When processing drugs/polymer blends through electrospinning, preserving the bioactivity of drugs is another challenge. Drugs would be in direct contact with organic solvents, high electric charge, and even mechanical stress during electrospinning. Therefore, the bioactivity of drugs, some labile molecules in particular, will be hindered. To stabilize the drugs, binding them to other molecules may be an option.^61^ For example, emulsion electrospinning of BMP-2 and PLGA resulted in denaturation of BMP-2.^67^ When included hydroxyapatite into the formulation, the bioactivity of BMP-2 was retained. Similarly, using bovine serum albumin (BSA) as a stabilizer for electrospinning of NGFs and poly (caprolactone-co-ethylethylene phosphate) led to preserved bioactivity to some extent.^68^ Blending drugs with polymers (e.g., silk fibroin) that can be electrospun under mild processing conditions can be another approach. Near-field electrospinning exhibits concentrated electric field; thus, it allows for a substantially reduced applied voltage. It can be further investigated for drug delivery since it can minimize the electrically charged effect on bioactivity. Coaxial electrospinning of drugs and polymers provides a more sustained drug release. Normally, drugs are embedded in the core and released through either pores in the sheath or shell polymer degradation. Due to the barrier effect of the sheath structure, an initial burst release can be avoided. The shell polymer solution guarantees the successful fiber formation in coaxial electrospinning, and the electric charge is predominantly found on the sheath.^61^ Therefore, core-sheath nanofibers are preferred for delivery of labile molecules such as enzymes and growth factors even cells can be included.^69^

One current research focus is on the construction of a “smart” drug delivery system for controlled release in terms of target-specific and triggerable. Incorporating drug loaded nanoparticles and microspheres into electrospun nanofibers is a common strategy for better control of drug release. The host fiber characteristics as well as the particle properties can be both tailored for specific requirements. The drug delivery system containing drugs encapsulated particles and electrospun nanofibers is highly desired in cancer therapeutics. Localized and controlled chemotherapy can be achieved with higher therapeutic efficiency but lower drugs dosage; hence, there is lower toxicity to the surrounding tissue. A pH-sensitive anti-tumor drug delivery system was constructed by encapsulating CaCO_3_-capped mesoporous SiO_2_ nanoparticles in PLLA electrospun fibers.^70^ Doxorubicin (DOX), an anti-tumor drug, was loaded into nanoparticles. The reaction between CaCO_3_ and the acidic environment on the tumor site would stimulate the release of DOX, while in the normal tissue with the physiological pH, only a minor release of such anti-tumor drug was detected. That pH-responsive anti-cancer efficacy can last over 40 days. Trametinib loaded hollow copper silicate was introduced to the PCL/poly (D, L-lactic acid) (PDLLA) electrospun matrix for chemo-photothermal therapy of melanoma.^71^ This anti-tumor drug delivery system was near-infrared irradiation (NIR)-sensitive. Releasing of multiple drugs attributes to a synergistic therapy that exhibits better antitumor efficacy. For example, co-delivery of paclitaxel and RNA interference (RNAi) to brain tumor simultaneously inhibited tumor angiogenesis and suppressed tumor cells proliferation.^72^ The RNAi and drugs were loaded in PEI nanoparticles and then encapsulated into PLGA electrospun microfibers. Surface immobilized of nanofibers or particles with functional groups allow for higher tumor cells affinity; hence, there is better target efficiency. In one demonstration, folate decorated PCL/PEG micelles were loaded with DOX and processed through coaxial electrospinning with PVA aqueous solution.^73^ DOX contained micelles resided in the core, while PVA nanofiber composed the outer sheath. Folate can bind to folate receptors that usually overexpressed on a number of solid tumors, which constructs an active-targeting drug delivery system.^74^

## BIOSENSORS AND CANCER DIAGNOSIS

**FIG. 6.**
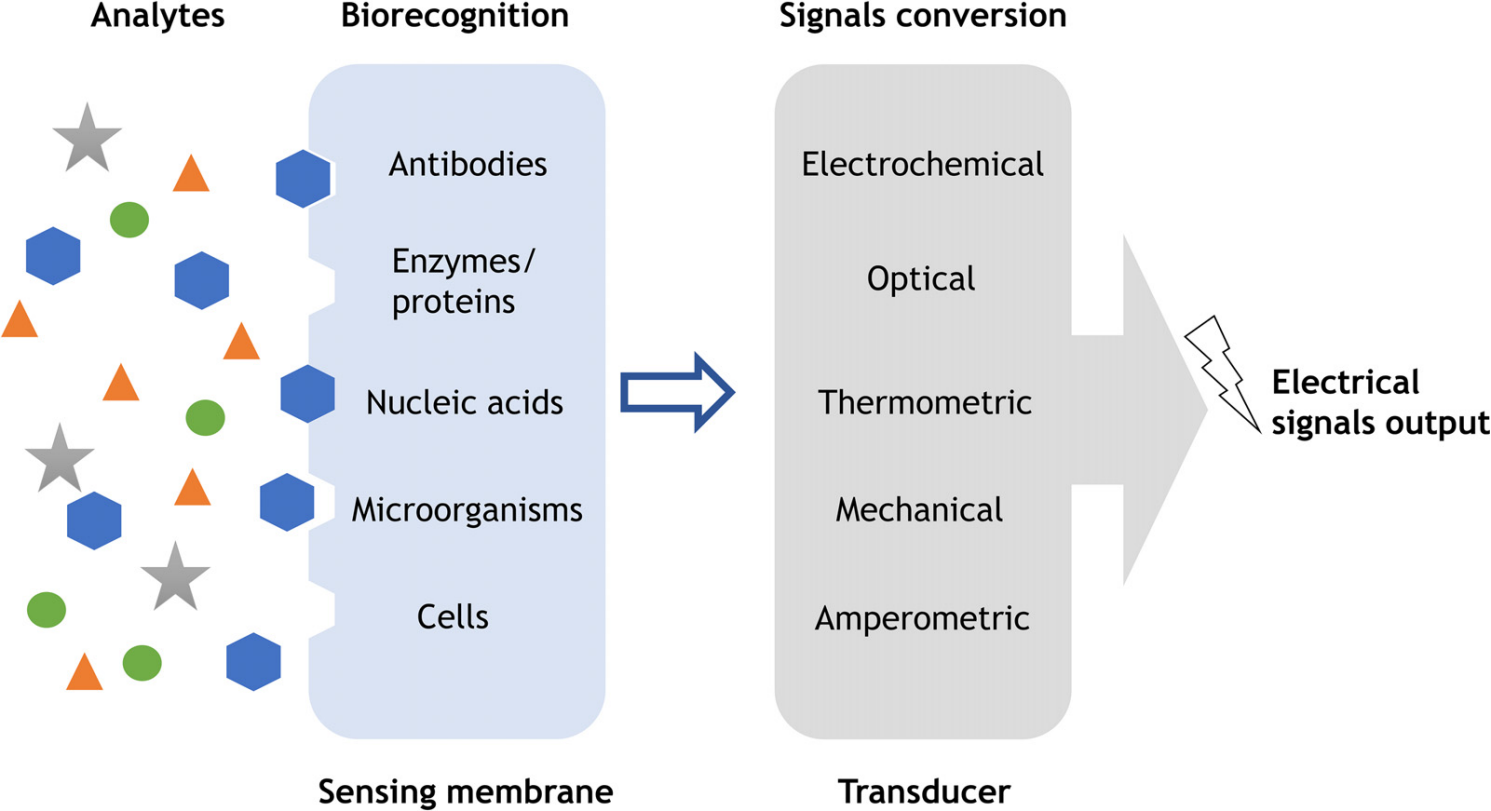
Schematic illustration of basic concept of a biosensor. Biorecognition elements such as antibodies, enzymes, and nucleic acids are incorporated in the sensing membrane to detect analytes via specific binding mechanisms. The transducer will convert acquired signals such as chemical substances, light, heat, etc., to electrical signals for further processing and analysis.

Biosensors are normally composed of biofunctional membranes and transducers for biological substances detection, where the sensing membrane is responsible for substances recognition, and transducer converts it into output signals^75^ (Fig. 6). The sensing membrane affects the performance of a biosensor including sensitivity, selectivity, reproducibility, and response time. High sensitivity is highly important in successful detection of biological substances at relatively low concentrations, to which electrospun nanofibers could contribute. The large surface-to-volume ratio of nanofibers allows more binding sites for analytes recognition; thus, it ensures an optimal sensitivity of electrospun nanofibers incorporated biosensors. Electrospun nanofibers are typically integrated into biosensors through two avenues.^76^ Electrospun functional polymers such as PANi can be directly used as the inducing element in biosensors. The other one is using electrospun nanofibers as templates for sensing materials deposition.

Incorporation of biomolecules to electrospun nanofibers is common for substance recognition. For example, glucose oxidase is a glucose sensitive enzyme, which has been widely immobilized on electrospun nanofibers for glucose biosensors construction. However, use of enzyme is usually associated with instability and denaturation problems. Therefore, several studies investigated non-enzymatic glucose sensing using carbon, metals, and their oxides as electrodes. In one demonstration, bimetallic nanoparticles CuCo doped electrospun carbon nanofibers were developed for enzyme-free glucose detection.^77^ Such glucose biosensor showed a high sensitivity, fast response within 2 s, long-term stability, and excellent anti-interference to electroactive molecules.

Electrospun nanofibers incorporating biosensor can be used for early cancer diagnosis. Substances to be detected for early cancer diagnosis include specific biomarkers overexpressed by cancer cells, oxygen level, and circulating tumor cells (CTCs). Specific antibodies, surface functional groups, and oxygen sensitive materials can be incorporated to electrospun nanofibers early diagnosis. For instance, epidermal growth factor receptor 2 (ErbB2)-antibody was conjugated to mesoporous ZnO nanofibers for breast cancer diagnosis.^78^ Combination between electrospun nanofibers with microfluidic techniques can improve the diagnostics sensitivity and CTC capture efficiency. A microfluidic immunosensor was designed for ErbB2 detection,^79^ which is a breast cancer biomarker. The immunoelectrode of this biosensor composed of the porous graphene foam modified with electrospun carbon-doped TiO_2_ nanofibers. This microfluidic biosensor allowed a wide concentration range of target ErbB2 antigen and achieved femtomolar sensitivity. CTCs are cancer cells that shed from the primary tumor and circulated into the bloodstream leading to metastasis.^80^ A microfluidic chip was fabricated for melanoma CTCs capture via conjugation of anti-146 antibody, a melanoma-specific capture agent, to PLGA nanofibers.^81^ This biosensor not only exhibited high CTCs capture efficiency, but also enabled specific isolation of single circulating melanoma cells.

## OTHER APPLICATIONS

### Medical device and implant coating

Electrospun nanofibers can be deposited on medical implants to improve the biocompatibility or acquire additional function. Nanofibrous coating would change the implant surface topography, thus providing topographical cues to surrounding cells and tissue. Incorporating bioactive agents or even cells into nanofibers can achieve multifunctional medical device coating such as drug delivery. Palumbo *et al.* coated biodegradable magnesium prosthesis with a porous PCL layer through electrospinning.^82^ Such porous coating could enhance cell adhesion and act as a drug releaser for anti-infection. A commercial coronary stent system, PK Papyrus (Biotronik),^83^ incorporates polyurethane as a stent coating for higher bending flexibility. A stent for aneurysm treatment was coated with poly (L-lactide-co-ε-caprolactone) (PLCL) nanofibers to achieve anti-coagulation and rapid endothelialization.^84^ Heparin and vascular endothelial growth factors were encapsulated into the core of PLCL nanofibers. Such core-sheath structure allows a sustained release for 30 days without an initial burst release. The nanofibrous coating can act as an interphase between the implant and host tissue to minimize the stiffness mismatch at the interface, which is quite critical in hard tissue prosthesis failure.^60^

### *In vitro* 3D cancer models

The *in vitro* cancer models provide an effective way for drug screening, anti-cancer mechanism, and tumor cell biology. Traditional 2D cancer models employ culturing tumor cell monolayer on 2D flasks, which can reflect cancer cell behavior to a certain degree.^85^ However, tumor cells behave differently in 2D models and 3D models. 2D cancer models are difficult to mimic cell-cell and cell-matrix interactions, whereas 3D models can offer a comprehensive understanding of the collective behavior between cells and cell-matrix, thus allowing more accurate control of tumor activities.^80^

Electrospun nanofibers mimic the native structure and composition of ECM for target tissue regeneration or mimic the stem cell niches for stem cell differentiation. Similarly, to construct an *in vitro* 3D tumor model, electrospun nanofibers play a role in replicating native extracellular environment for the tumor cells. Electrospun nanofibers can realize the biochemical stimuli of native tumor ECM through surface functionalization with biomolecules or drug encapsulation. For instance, perlecan domain IV peptide was conjugated on electrospun PCL/gelatin nanofibers to construct a pharmacokinetic prostate cancer model.^86^ Tumor ECM yet exhibit a physiological complexity and heterogenous feature.^80^ Currently, electrospun *in vitro* 3D tumor models encounter some challenges including failure in mimicking the tumor angiogenesis, heterotypic cell-cell signaling, biomechanical stimuli, and insufficient cell infiltration. Inclusion of bioreactors in model fabrication can provide mechanical stimulus to cancer cells.^87^ Combining electrospinning with bioprinting technology may help with more sophisticated 3D cancer models development.

### Filtration membranes for biomedical applications and wearables

Large surface-to-volume ratio, light weight, high porosity, interconnectivity, and microscale interstitial space make electrospun nanofiber meshes an excellent material for filtration applications in terms of personal protective equipment. Conventional protective clothing is based on full barrier protection that is limited in weight and moisture retention.^88^ Electrospun nanofibers can contribute to light-weight and breathable fabrics production that permit air and water vapor transport but filter other undesired agents.^60^ Protective masks are typical used filtration membranes in biomedical applications. Chemical and biological threats such as nerve agents, bacteria, and virus can be blocked and even decomposed through active reagents embedded on the nanofibrous membrane. Electrospun nonwoven PAN nanofibrous mats incorporated with sliver nanoparticles were used for bacterial filtration.^89^ Such filter possessed 99% bacterial filtration efficiency with promising anti-bacterial activity. There is supply shortage of face masks during the outbreak of COVID-19. Researchers from Korea Advanced Institute of Science and Technology (KAIST) developed washable and reusable nanofiber filtered masks to deal with it.^90^ The masks contain electrospun orthogonally aligned nanofibers with a diameter of 100–500 nm. Upon 20 repeated bactericidal tests with ethanol, masks maintain more than 94% filtration efficiency and are water resistant without deformation in the membrane structure. Inovenso Ltd. fabricated nanofiber face masks containing non-woven electrospun layers with 99.9% filtration efficiency.^91^ It is believed that nanofiber masks would be the new generation with their small pore size, large surface area, and light weight.

## CONCLUSION

We overviewed the advances of electrospun nanofibers in biomedical applications and highlighted the application in tissue engineering, drug delivery, biosensors, and cancer diagnosis. There are various types of commercial biomedical products based on electrospun nanofibers (Table III). Electrospinning allows the manipulation of material properties down to the nanoscale, which is the most consistent scale to native ECM. Instead of the traditional 2D scaffolds used in those applications, 3D scaffolds are more favored for more efficient resemblance to native ECM. A complete replication of cell-matrix interactions can enhance the regeneration efficiency. Therefore, the influence of nanofibers on cellular signaling mechanisms and biochemical pathways is encouraged to further investigate for a better understanding of cell-nanofiber interactions. Drugs can be encapsulated into nanofibers through post-electrospinning modifications, blend electrospinning as well as coaxial electrospinning, and nanoparticles incorporation. Different drug loading methods result in different drug releasing profiles. Drug releasing from nanofibers following a predictable spatiotemporal profile is more expected in the future, whereas there are a few studies of pharmacokinetics of drug-loaded electrospun nanofibers, which would limit the practical application. Overall, the future opportunities would lie in multifunctional electrospun products development based on combination of various applications. For example, drug releasing nanofibrous scaffolds can simultaneously promote tissue regeneration and achieve therapeutic function or anti-tumor drug embedded biosensors for early cancer diagnosis and chemotherapy. Combining electrospinning with other biofabrication techniques can benefit 3D hierarchical and heterogenous structure construction such as cooperation with microfluidic technique for cancer biosensors and with bioprinting for 3D cancer models.

**TABLE III.** Examples of commercial electrospun products for healthcare. β-TCP: β-tricalcium phosphate; SiV: siloxane-containing calcium carbonate.

Commercial products	Company and country	Stage of products	Electrospun material	Application
AVflo™	Nicast (Israel)	CE certified (2008)	PU	Nanofibrous vascular grafts
Bioweb™	Zeus (USA)	Clinical use	PTFE	Vascular stent coating
PK Papyrus	Biotronik (Germany)	FDA approved (Sept. 2018)	PU	PU covered coronary stent system
ReBOSSIS	ORTHOREBIRTH (USA)	FDA cleared (2018)	β-TCP/PLLA/SiV	Bone void filler
SurgiClot^®^	St Teresa Medical (USA)	Clinical use	Dextran	Hemostatic wound dressing
ReDura™	MEDPRIN (Germany)	Clinical use	PLLA	Dural substitute patch
NeoDura™	MEDPRIN (Germany)	Clinical use	Synthetic polymers /gelatin	Dural substitute patch
Rivelin^®^ patch	Bioinicia (Spain)	Clinical trial (phase 2)	Drug delivery layer: PVP/Eudragit RS100^®^ Hydrophobic backing layer: PCL	Mucoadhesive drug delivery patch
HealSmart™ personalized antimicrobial dressings	PolyRemedy^®^ (USA)	Clinical use	Hyaluronic acid	Personalized wound-care system
SpinCare™	Nanomedic (Israel)	CE certified (2017)	…	A portable bedside wound care device
Stem cell culture/extract sheet	ORTHOREBIRTH (USA)	…	Bioresorbable polymers	Stem cell culturing for research studies
BioPaper™ Technology	Dipole Materials (USA)	…	Various materials (e.g. gelatin, colagen)	Lab tissue culture
Cytoweb^®^ sheets	eSpin (USA)	…	PLGA, PLA, PCL, PU	*In vitro* cell culture
NanoAligned™	Nanofiber solutions (USA)	…	PCL	3D cell culture
Mimetix^®^	Electrospinning Company (UK)	…	PLLA	Multi-well plates for 3D cell culture

By optimizing properties, nanofibrous mats in terms of fiber diameter, orientation, morphology, porosity, mechanical properties, and electrospun nanofibrous products can be explored for more specific requirements of different applications. Melt electrospinning and near-field electrospinning can avoid toxic solvents incorporation and high electric field interference, respectively. Inspired by AM, direct writing electrospinning allows development of highly customizable 3D constructs with well-defined features, while those techniques typically generate fibers on micrometer scale, which limits their employment in nanometer scale favored applications. Hence, a complete understanding of electrospinning mechanisms, specifically in the fast acceleration zone that contributes to sufficient thinning of fibers, will benefit the prediction of fiber properties. Simulation and modeling design of such mechanism can help with electrospinning parameters selection prior to the actual experiments. There are concerns associated with the environmental effect of organic solvents during solution electrospinning. How to achieve a “green” process remains to be addressed. Besides, there are difficulties in scale-up production due to the lack of a reliable system for quality control. With a comprehensive understanding of the electrospinning mechanism and better control of nanofiber properties, further developments of electrospinning in healthcare in the following decades can be expected.

## Data Availability

The data that support the findings of this study are available from the corresponding author upon reasonable request.

## NOMENCLATURE

PCL: poly(ε-caprolactone)
PLLA: poly(L-lactic acid)
PDLLA: poly(D, L-lactic acid)
PLA: poly(lactic acid)
PLGA: poly(lactic-co-glycolic) acid
PLCL: poly(L-lactide-co-ε-caprolactone)
PAN: polyacrylonitrile
PANi: polyaniline
PEI: polyethylenimine
PEG: polyethylene glycolPVApoly(vinyl alcohol)
PU: polyurethane
PVP: polyvinylpyrrolidone
PEO: poly(ethylene oxide)
